# Partial Storage Optimization and Load Control Strategy of Cloud Data Centers

**DOI:** 10.1155/2015/836561

**Published:** 2015-04-20

**Authors:** Klaithem Al Nuaimi, Nader Mohamed, Mariam Al Nuaimi, Jameela Al-Jaroodi

**Affiliations:** ^1^UAE University, P.O. Box 15551, Al Ain, UAE; ^2^University of Pittsburgh, Pittsburgh, PA 15260, USA

## Abstract

We present a novel approach to solve the cloud storage issues and provide a fast load balancing algorithm. Our approach is based on partitioning and concurrent dual direction download of the files from multiple cloud nodes. Partitions of the files are saved on the cloud rather than the full files, which provide a good optimization to the cloud storage usage. Only partial replication is used in this algorithm to ensure the reliability and availability of the data. Our focus is to improve the performance and optimize the storage usage by providing the DaaS on the cloud. This algorithm solves the problem of having to fully replicate large data sets, which uses up a lot of precious space on the cloud nodes. Reducing the space needed will help in reducing the cost of providing such space. Moreover, performance is also increased since multiple cloud servers will collaborate to provide the data to the cloud clients in a faster manner.

## 1. Introduction

**Figure 1:**
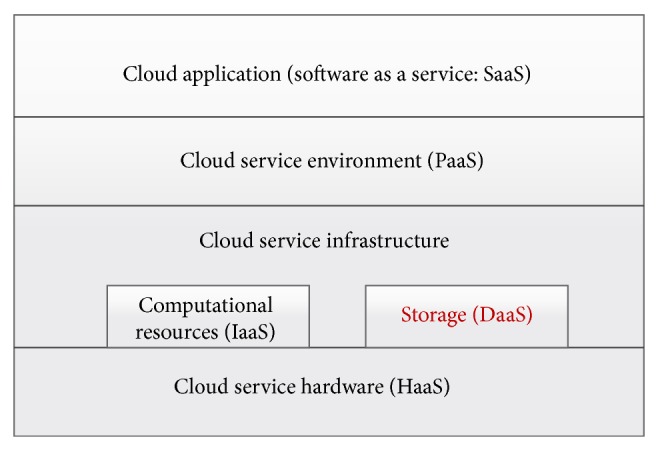
Cloud services structure.

The cloud is emerging strongly and offering technology solutions that are widely used around the world. Generally, there has to be some type of cloud utilization within most IT infrastructures of organizations. The cloud provides a variety of services for its users [1] such as providing software as a service (SaaS), infrastructure as a service (IaaS), and platform as a service (PaaS). Providing infrastructure as a service within the cloud includes providing data (DaaS) or computational resources services (see Figure 1). There are many areas that can be improved in the cloud services such as the security of the data, the reliability of the cloud services, and the usage of distributed third party servers without including the client in the back-end processes [2]. Moreover, a problem that still needs to be addressed for providing DaaS in the cloud is improving the techniques used to make the data available and quickly accessible (and downloadable) for the client. Another problem is data redundancy on the cloud servers, which is resulting in a huge increase of storage needs. Recently, various research projects are being done to find effective and efficient solutions for both storage problems [3, 4].

Our focus in this paper is on how to improve DaaS on the cloud by enhancing the speed by which data is fetched from the cloud servers and provided to the users. Moreover, we focus on optimizing the storage needed from each cloud server by our novel technique using data partitioning [5–7]. We try to reduce the data redundancy on the cloud servers to improve the processing and reduce the cost needed from the cloud providers. We extend our previous work and provide improved results. We also compare our technique with other well-known cloud load balancing techniques in terms of processing speed and storage optimization.

The rest of this paper is organized as follows. We discuss the background and current literature of load balancing and storage optimization in the cloud in Section 2. In Section 3 we discuss our original dual direction download which is a base for our work in this paper. After that, we provide a detailed view on how our solution is enhanced and the overall architecture of the solution in Section 4. We then show simulation results of our approach in Section 5. Finally, we conclude with Section 6.

## 2. Background and Related Work

In this section, we first provide some background information about DaaS and discuss the motivation behind our research and the current problems facing providing DaaS in the cloud. We then provide an analysis of the current approaches discussed in the literature and highlight the areas we focus on to enhance in our approach.

### 2.1. DaaS Background

**Figure 2:**
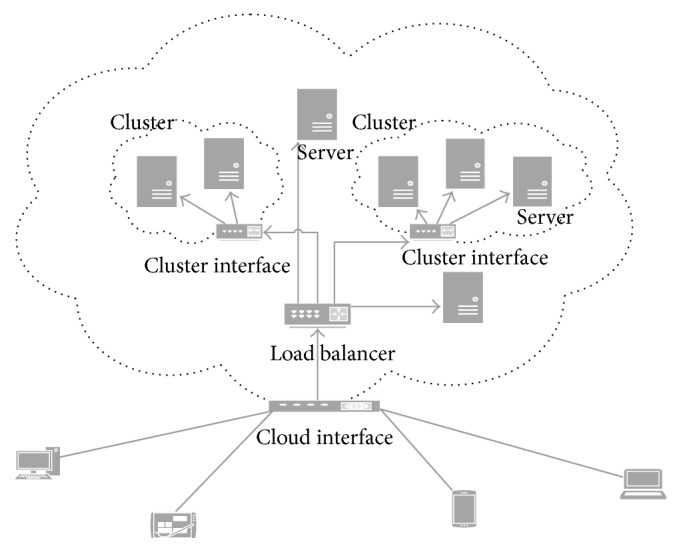
DaaS structure.

DaaS [8] as mentioned earlier stands for data as a service. Providing data as a service on the cloud means enabling organizations to provide some common and needed data for different cloud users. This data is made available on some cloud servers. Generally back-end processes of assigning tasks to servers and downloading data from them is hidden from the end users because it is not of interest for them. DaaS is also viewed in [9] as providing data in different formats for different resources in various geographical locations. The resources would be able to upload, download, and edit the data on the cloud based on their assigned privileges. Usually, the cloud has multiple distributed servers which are able to access the data centers and fetch the data from them.  Figure 2 shows how the cloud DaaS is usually structured.

### 2.2. Research Motivation

Cloud services have become a trend in the last decade because of their agility, location independence, and cost effectiveness. There are many organizations and cloud providers that offer DaaS services. These are very common services among users and are a very reliable solution to keep large files and share them. Examples of the most well-known industry applications are Dropbox, Google Drive, Apple iCloud, and Microsoft OneDrive. The services provided by each of the mentioned applications vary from providing the ability to upload and share files to the amount of storage provided to the client.  Table 1 shows a comparison of the most well-known applications in the industry [10]. It is found that free storage provided to normal users range from 2 GB to 15 GB. However, the premium storage can reach up to 200 GB. Dropbox application is the dominant application in the market with 47.9% market share.

Dropbox announced recently that the number of their users reached 270 million [11]. Imagine having at least 2 GB for 270 million users. The problem here is that storage consumes most of the cost spent to provide DaaS. As stated by Greenberg et al. [12] in his analysis of cloud costs, data centers consume 45% of the total costs, infrastructure consumes 25% while networks, and power draw consumes 15% each. Therefore, there is a strong need for efficient cloud-based storage. The storage utilization, however, must not negatively affect the download speed at the client side or the reliability and availability of the storage. The main focus of this paper is to offer an effective and efficient load balancing technique to enhance the download performance and optimize the storage usage for DaaS on the cloud.

### 2.3. Related Work

In this section we discuss current contributions in the literature for load balancing and storage optimization on the cloud. We classify the load balancing algorithms into two types: static algorithms and dynamic algorithms. Most of the work done in the literature is either to enhance the performance or the storage consumption, rarely addressing both. Most of the related work which focuses on enhancing the performance usually deals with enhancing the load balancing algorithm. However, this may lead to a more space usage, while approaches addressing the space issues may negatively affect the overall performance.

#### 2.3.1. Static Load Balancing Algorithms

Static load balancing algorithms assign the tasks to the nodes based only on the ability of the node to process new requests. The process is based solely on prior knowledge of the nodes' properties and capabilities. These would include the node's processing power, memory and storage capacity, and most recent known communication performance. Although they may include knowledge of the communication prior performance, static algorithms generally do not consider dynamic changes of these attributes at run-time; thus, they cannot adapt to load changes.

Radojević and Žagar suggested an algorithm called CLBDM (Central Load Balancing Decision Model) [13]. CLBDM is an improvement of the Round Robin Algorithm which is based on session switching at the application layer. Round Robin [14] is a simple and effective load balancing algorithm. However, it sends the requests to the node with the least number of connections. The improvement done in CLBDM is that the connection time between the client and the node on the cloud is calculated, and if that connection time exceeds a threshold then there is an issue. If an issue is found, the connection will be terminated and the task will be forwarded to another node using the regular Round Robin rules. CLBDM acts as an automated administrator inspired by the human administrator point of view.

The proposed algorithm by Nishant et al. [15] is an improved version of the algorithm presented in [16]. Both algorithms use the ants' behavior to gather information about the cloud nodes to assign a task to a specific node. However, the algorithm in [16] has the ants' synchronization issue, which is addressed in [15] by adding the feature “suicide.” Both algorithms work in the following way, once a request is initiated the ants and pheromones are initiated and the ants start their forward path from the “head” node. A forward movement means that the ant is moving from one overloaded node looking for the next node to check if it is overloaded or not. Moreover, if the ant finds an underloaded node, it will continue its forward path to check the next node. If the next node is an overloaded node, the ant will use the backward movement to get to the previous node. The modification proposed in [15] forces the ant to commit suicide once it finds the target node, which will prevent unnecessary backward movements.

The algorithm proposed in [17] is an addition to the MapReduce algorithm [18]. MapReduce has two main tasks: it maps tasks and reduces tasks results. Moreover, there are three methods “part,” “comp,” and “group” in this model. MapReduce first executes the “part” method to initiate the mapping of tasks. At this step the request entity is partitioned into parts using the Map tasks. Then, the key of each part is saved into a hash key table and the “comp” method does the comparison between the parts. After that, the “group” method groups the parts of similar entities using the Reduce tasks. Since several Map tasks can read entities in parallel and process them, this will cause the Reduce tasks to be overloaded. Therefore, [17] proposes to add one more load balancing level between the Map task and the Reduce task to decrease the overload on these tasks. The load balancing addition divides the large tasks into smaller tasks and the smaller blocks are sent to the Reduce tasks based on their availability.

Ni et al. proposed a load balancing algorithm for the private cloud using virtual to physical machine mapping [19]. The algorithm contains a central scheduling controller and a resource monitor. The scheduling controller does all the work for calculating which resource is able to take the task and then assigning the task to that specific resource. However, the resource monitor does the job of collecting the details about the resources' availability. The process of mapping tasks goes through four phases: accepting the virtual machine requests, getting the resources details using the resource monitor, calculating the resources ability to handle tasks (from the clients' requests) and assigning the resources with the highest scores to the tasks, and providing access to the application for the client.

#### 2.3.2. Dynamic Load Balancing Algorithms

Dynamic load balancing algorithms take into account different attributes of nodes capabilities and network bandwidth. These algorithms assign and sometimes reassign the tasks dynamically to the nodes based on the attributes calculated before and during run-time. Such algorithms are usually harder to implement and may impose additional overhead; however, they are more efficient and provide better overall results.

Ren et al. [20] presented a dynamic load balancing algorithm for cloud computing based on an existing algorithm called WLC (weighted least connection) [21]. The WLC algorithm assigns tasks to the node based on the number of connections that exist for that node. This is done based on a comparison of the sum of connections of each node in the cloud and then the task is assigned to the node with the least number of connections. However, WLC does not take into consideration the capabilities of each node such as processing speed, storage capacity, and bandwidth. The proposed algorithm is called ESWLC (Exponential Smooth Forecast based on Weighted Least Connection). ESWLC improves WLC by taking into account the time series and trials. ESWLC builds the decision based on an experience of node's CPU, memory, number of connections, and load of disk occupation. ESWLC then predicts the node to be selected based on exponential smoothing.

The algorithm proposed in [5–7] is a dual direction downloading algorithm from FTP servers (DDFTP). The algorithm presented can be also implemented for load balancing data downloads on the cloud. DDFTP works by splitting an *m* long file into *m*/2 partitions. Then, each server node starts processing the task assigned for it based on a certain pattern. For example, one server will start from block 0 and keeps downloading incrementally while another server starts from block *m* and keeps downloading in a decrementing manner. Finally, when the two servers download two consecutive blocks, the task is considered finished and other tasks can be assigned to the servers. The algorithm reduces the network communication needed between the client and nodes and therefore reduces the network overhead. Moreover, attributes such as network load, node load, and network speed are taken into consideration.

The paper [22] proposes an algorithm called Load Balancing Min-Min (LBMM). LBMM has a three-level load balancing framework and uses the Opportunistic Load Balancing algorithm (OLB) [23]. OLB is a static load balancing algorithm that has the goal of keeping each node in the cloud busy. However, OLB does not consider the execution time of the node. This might cause the tasks to be processed in a slower manner and will cause some bottlenecks since requests might be pending waiting for nodes to be free. LBMM improves OLB by adding a three-layered architecture to the algorithm. The first level of the LBMM architecture is the request manager which is responsible for receiving a task and assigning it to one service manager in the second level of LBMM. When the service manager receives the request, it divides it into subtasks to speed up the processing of that request. A service manager also assigns the subtasks to service nodes that are responsible for executing the subtasks. The service manager assigns subtasks to the service nodes based on attributes such as the remaining CPU space (freeness of the nodes), remaining memory, and the transmission rate.

#### 2.3.3. Storage Optimization Techniques

Various aspects of storage optimization have been studied to reduce the space usage while maintaining consistent, reliable, and highly accessible data. The following are some examples of this research pertaining some level of relevance to our work.

The INS (Index Name Server) algorithm minimizes data duplication and redundancy [24]. INS integrates deduplication and access point selection optimizations. Several parameters are involved in calculating the optimum selection point. Some of these parameters are the hash code of the block of data to be downloaded, the position of the server that has the target block of data, the transmission quality calculated based on the node performance and a weight judgment chart, and the maximum bandwidth available for download from the target server. Another calculation is used to specify whether the connection can handle additional nodes (busy level). The busy level is classified in 3 categories B(a), B(b), and B(c). B(a): the connection is very busy and cannot handle more connections. B(c): the connection is not busy and additional connections can be added. B(b): the connection is limited and there is a need for further study. B(b) is further divided to 3 categories: B(b1): INS must analyze and establish a backup, B(b2): INS must send the requests to the backup nodes, and B(b3) (highest level of efficiency required): INS must reanalyze and establish new backups.

Zhang et al. [25] proposed a full replication solution that targets the download of small files from the cloud. The solution is referred to as BerryStore. The targeted files' maximum size is 10 MB. The advantage of this solution is to group many small files into one large file for which there is only one directory entry in the cloud nodes. This will minimize the search and queries of the small files where there will be only one query method for all files. The main structure of the solution is the client, NameServer, and DataServer. The client requests the file, the NameServer gets the location of that file (in which large file it is located), and the DataServer contains the real file data from which the client can download the actual file. The solution is good, yet it is not practical for large files. Moreover, the solution replicates the grouped large files on multiple cloud nodes which also can be enhanced by reducing the replication times.

Srivastava et al. [26] proposed another solution that works for single and multicloud storage. It reduces the efforts needed to migrate the client data from one cloud to another. Each cloud contains multiple clusters, VMs, and physical servers. Therefore, for each cloud there will be a CloudInterface and for each cluster there will be a ClusterInterface. The purpose of interfaces is to organize the interactions between the clients and the clusters within the cloud. Moreover, there is a broker that gets the client's request and processes it to the multiclouds. The client submits requests to the broker to either upload or download. For an upload request, the client specifies the SecurityLevel to be used by the FileSplittingFunction to split the file into multiple files based on the percentage of SecurityLevel provided by the client. For example, if the client specifies SecurityLevel to be 50%, then the file will be split into two subfiles each saved in a different location. For each cloud, the number of subfiles is equal to the number of free virtual machines. The limitation of this approach is its complexity, especially when the files are saved on different clouds.

Villari et al. [27–29] proposed Redundant Residue Number System (RRNS). Their main concern is the security of the client files hosted on the cloud. It is similar to Srivastava's solution. However, it is different as it keeps the metadata of the partitions and their locations on the cloud with the client as an XML file. This increases security as the only one who can collect all the partitions and create the original file is the client. The solution is also useful for clients dealing with multicloud providers. The number of file partitions and the Redundancy Degree (RD), which refers to the number of replications of the partitions in each cloud node, are specified by the client. The solution has 4 phases: splitting, dissemination, Retrieval, and reconstruction. The problem here is that if the client lost the metadata file, he/she will not be able to download the file. Moreover, file chunks are saved as XML files; thus, more processing is needed to convert the files to their original formats.

## 3. Discussion

The different approaches of load balancing offer specific solutions that suit some situations and not others. The static algorithms are usually very efficient in terms of overhead as they do not need to monitor the resources during run-time. Therefore, they would work very well in a stable environment where operational properties do not change over time and loads are generally uniform and constant. The dynamic algorithms on the other hand offer more effective solutions that could adjust the load dynamically at run-time based on the observed properties of the resources. However, this leads to high overhead as constant monitoring and control will add more traffic and may cause more delays. Some newly proposed dynamic load balancing algorithms try to avoid this overhead by utilizing novel task distribution models.

Table 2 compares the reviewed algorithms in terms of the challenges discussed in Section 2. For example, the only algorithm that avoids data redundancy and storage replication is the INS algorithm. However, INS is centralized and therefore has a single point of failure. Moreover, it is a complex algorithm. DDFTP relies on replicated resources and does not reduce the required storage space; however, it has a dynamic decentralized approach. It is also a much simpler algorithm to download stored data from replicated sources. Generally, each algorithm satisfies a partial set of these challenges, which makes it suitable for specific situations. For example, INS, CLBDM, and VM mapping are all efficient, yet they all have a single point of failure. As a result, they would function very well in a stable environment with very reliable resources. Moreover, all algorithms except for the Ants Colony and VM mapping can handle highly distributed environments. Therefore, they are more suitable for the public cloud. In addition, all but DDFTP introduce high overhead on the network. As a result, if the network conditions worsen, they would all suffer significantly and will affect the overall performance of the solution. However, DDFTP would be more capable in handling such delay as it does not rely on run-time monitoring and controls.

When comparing the storage optimization techniques, we noticed that each is focusing on a different aspect of enhancing the storage consumption. Some would look for reducing the number of small files in the cloud to have a better file structure and smaller number of files on the cloud like BerryStore. While other works like RRNS focus on enhancing the security of the files and therefore scatter file fragments among multiple cloud servers and keep the metadata with the client. This is beneficial as a security measure allowing for higher safety controls for the client. However, if the client loses the metadata file, a serious issue would occur because no one else has the same information. Therefore, for most of the storage optimization approaches, performance was not the main consideration.

Through our study of the different related work, we noticed that most of the common approaches in load balancing usually replicate the full files in all cloud servers. The benefit of replicating the files in all servers is to distribute the effort among the cloud servers and offer better reliability, availability, and response times. However, the storage needed when replicating the full files in all servers is huge, especially when we focus on large size files such as data mining files, videos, and scientific research data files. Replicating a file of size one TB on 5 cloud servers, for example, will consume most of the cloud costs. Another limitation that we noticed is that most of the approaches assign the full file download to one server. The problem in this case is that on the Internet, the network load on that server's network and the server speed are not predictable. For example, if a server* S* at the assignment step was free and it was assigned to download the full file* X*. Then, server* S* network was down, or it was loaded, and this will affect the speed of downloading file* X* at the client side. Therefore, it is better to partition the file into multiple partitions and assign them as tasks to multiple servers to distribute the effort and resolve the issue of one server-one file download.

## 4. Proposed Solution

Here, we show our original dual direction download approach and then discuss the optimization to reduce the storage needs without affecting the performance.

### 4.1. Dual Direction Download Approach

**Figure 3:**
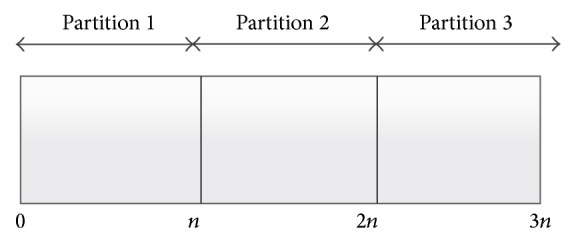
File partitioning for DDFTP download.

In this section we show how the original dual direction download works. The selected algorithm (DDFTP) [5, 7] is the dual direction file retrieval from the cloud servers. The algorithm works by splitting the file into partitions of data as shown in Figure 3 and assigning two cloud servers for each partition to download the data from opposite directions. Each of the cloud servers will handle a download of either forward or backward in the partition depending on its assignment. This way, the download is parallelized across the available replicas and the overall client download time is improved significantly. In addition, the approach provides an efficient load balancing scheme with minimum coordination and zero interaction among the servers being used. However, DDFTP requires the existence of full replicas of the data set on each of the cloud server nodes in use.

**Figure 4:**
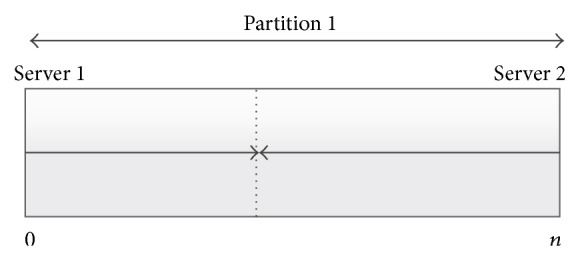
Download directions of two cloud servers.

If we assume that each partition is of length *n*. Then, for each two cloud servers, the first server will provide the data starting from block 0 and increment its counter to move forward, while the second will provide the data starting from block *n* − 1 and decrement its counter to move backwards as shown in Figure 4.

The algorithm works well for file download and shows good performance [5]. However, data storage is still consuming a lot of space on each cloud server and the same data files are saved on each server. Although some parts of these replicas never get used. This means that the storage consumption is more than needed and, therefore, our target is to reduce the storage consumption by improving the DDFTP algorithm and applying the partial replication of the data files on the cloud servers. This means that we will not store the same data file on all cloud servers. We will store different parts of the data files on each cloud server according to the servers' performance throughout the different times download requests were performed on the server.

### 4.2. Optimized Storage of Collaborative Cloud Servers Approach

In this section, we show our partial replication load balancing technique where we focus on improving the performance of task scheduling, processing, and storage of space optimization for DaaS.

**Figure 5:**
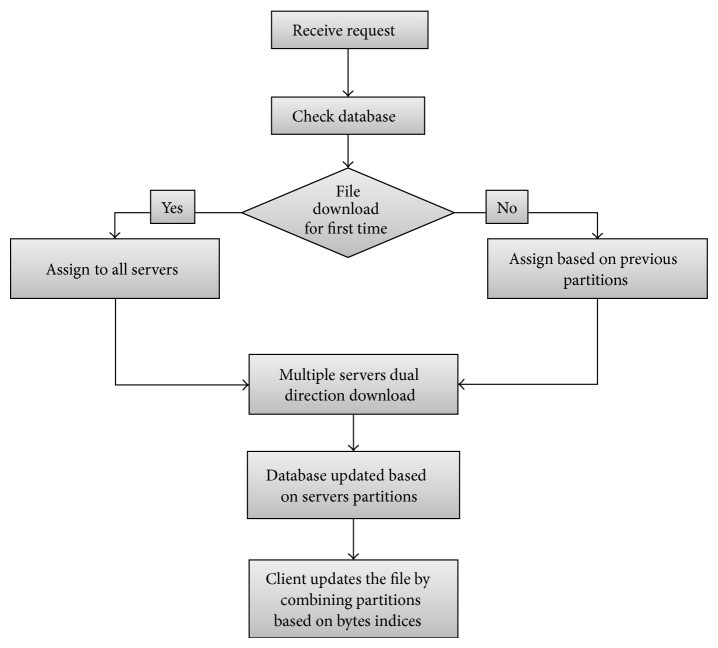
Downloading file from cloud workflow.

**Figure 6:**
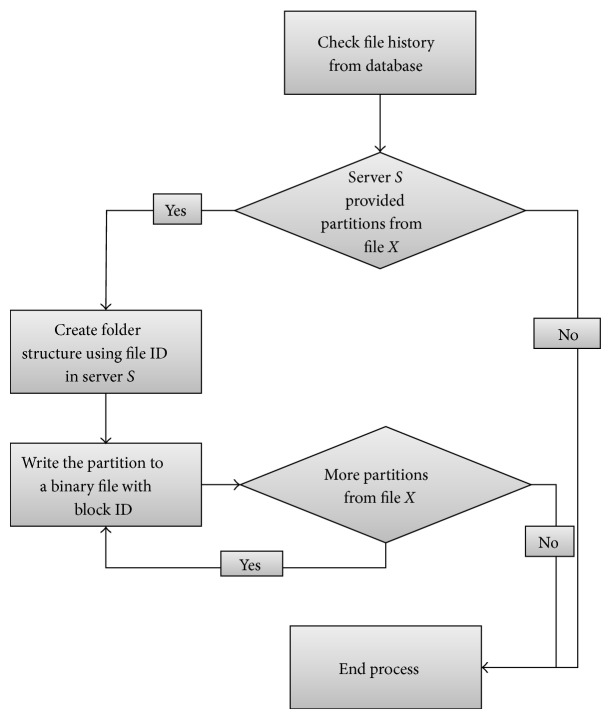
Cloud file redundant data removal process.

To implement this technique we used the workflows shown in Figures 5 and 6.  Figure 5 describes the workflow of downloading a file by the cloud client. To download a file, the client initiates a request to the cloud. The cloud controller then checks if the file was downloaded before; if so, then there will be data about the file partitions that were downloaded and which servers provided them. Having this history helps in selecting which server must provide which partition. The controller finds the required data from the database and then assigns the servers which already have the file partitions to the task. After the data is downloaded from all the servers, the client is updated by the requested file. However, there must be a first time download for each file to get its experience. Therefore, the alternative workflow is selected if the file is being downloaded for the first time. The file size in bytes is fetched and the block size is determined (Pseudocode 1). Then, servers are assigned based on their availability and processing speeds. When the dual direction download is processed from all servers for the first time, the client and the database are updated. The database must always be updated with what happens in the servers processing each partition so that the controller can decide later which partitions are to be kept on the server and which are to be removed.

We allow the file partitioning process at the controller side when the controller has enough data to make its decisions.  Figure 6 shows how the controller saves the required partitions on the servers and get rid of the redundant partitions based on their download rate. To do that, the controller first checks the available data in the database about the download from the previous servers' experiences. Then, if blocks downloaded from server* S,* for example, were found, the controller creates a directory in server* S* where the directory name is the file* X* ID. In the server folder, the blocks that were downloaded from that server are copied. Each block will be a file by itself and the name of the file will be the block ID. We tested splitting the original file into the blocks and combining them by the client. The original file was created at the client without any problems. Therefore, this could be the best way to keep partitions of the file in the server without the need for complicated calculations. The file sizes will be matching the block size in the original file.

To explain our technique, assume we have cloud servers* A*,* B*,* C,* and* D*, each of which has different performance, storage, and speed attributes. The first time the request to download file *X* is initiated, the controller will get the number of blocks within file *X* and split them into two partitions so that each two servers will be responsible for downloading one partition. Assuming that the number of replicas of the file *X* is *M*, and the file *X* has *N* blocks, then we need to divide the file into *M*/2 partitions. In addition, we can calculate the size of each partition as

(1) $$\begin{array}{c} \text{Partition}\;\text{Size}=\left(\frac{N}{M}\right)\times 2. \end{array}$$

Moreover, a block size must be decided based on the original problem size (file *X* size). To do that we factorize the original file *X* size and find the biggest factor that belongs to the interval from 0 which is the minimum file size to *X*/NOC∗NOS which refers to the file size divided by the maximum number of connections allowed by the database server multiplied by the number of servers. Having this interval will prevent any “*exceeding number of connection*” errors for the users when uploading their files to the cloud servers. Since we keep the metadata in the database then it is important to consider the database server's ability to receive update connections. It is also important to mention that NOC is fixed per database server. However, NOS changes according to the number of available servers when the file upload request was initiated. This number would be fixed per file after the upload process. *X* is also a variable that changes per file. Experiments to validate this equation are in Section 4:

(2) BlockSize=Max⁡fx:  wherefx∈0,xNOC∗NOS.

The pseudocode in Figure 6 shows how the block size is determined based on (2). The file size is first acknowledged. Then, the factorization method is applied and when the largest number in the required interval is found, it is updated in the BlockSize table in the controller. This is so that the block size is determined for each file when the file is first uploaded, while the number of blocks *N* in file *X* can be found by dividing the file *X* size *R* by the block size found in (2). The equation to find *N* is as

(3) $$\begin{array}{c} N=\frac{R}{\text{BlockSize}}. \end{array}$$

During our experience we found that the number of replicated blocks in more than one cloud servers is associated with the number of coordinated servers in the download. It is also associated with the load assigned to each server and the speed of the server. For example, if we had only two nodes downloading the file, and both nodes have the same load and the same speed, then the number of replicated blocks on the two servers from the file will be 2. While if the number of nodes downloading the file are 4, the number of replicated blocks will be 4. If one of the dual servers was faster than the other, then the number of replicated blocks could increase to 3. This is because one server processes the request faster than the other and for the other server to reach it, more blocks get replicated. We are working on finding out an optimization formula to calculate the number of blocks based on all given attributes.

Therefore, if we have four replicas of a file on four cloud servers then we need to divide the file into 4/2 = 2 partitions. If file *X* has 3000 blocks, for example, then each partition will be of size (3000/4)∗2 = 1500 blocks. Assuming we have servers* A*,* B*,* C,* and* D*, when the first request is initiated; the controller will look for the free servers and assign the partitions to them. In this example, partition 1 will be assigned to servers* A* and* B*. Server* A* will provide the forward download of partition 1 while server* B* will provide the backward download of the same partition. As the servers push the blocks they also update their blocks download counters as in Tables 1 and 2, where the partition is of size *P* and server* A* downloads from 0 onwards and server* B* downloads from *P* − 1 downwards until they meet at blocks *k* and *k* + 1.

Similarly the second partition is assigned to cloud servers* C* and* D* and they both keep similar tables. These tables are updated every time a download request is assigned to the servers for the same file. This will allow the servers to know which blocks are being used and which ones are not. Over time and with the repetitions of the downloads the servers can decide to remove the blocks that are never used from storage. This way if we examine servers* A* and* B* after a while we may find that server* A* has pushed blocks 0 to *k* at least once, while the remaining blocks in the partition were never used. In addition, server* B* has pushed blocks *P* − 1 to block* j* at least once while the others were never used. In this case, the controller may decide to tell server* A* to delete blocks *k* + 1 to *P* − 1 and server* B* to delete blocks 0 to *j* − 1. Assuming varying performance and loads on the two servers, *j* will usually be smaller than *k*; thus, there will be some overlap across the servers to ensure proper download in the upcoming requests. For this approach to work correctly, we must make sure that the downloads on particular servers are always done in the same direction. For example, server* A* will always be assigned to start from the beginning of a partition, while server* B* will always start from the end of the partition. The same applies to all servers participating in the download process.

**Figure 7:**
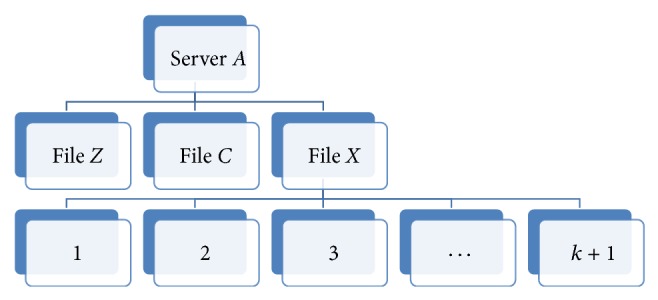
Cloud Node* A* file structure.

**Figure 8:**
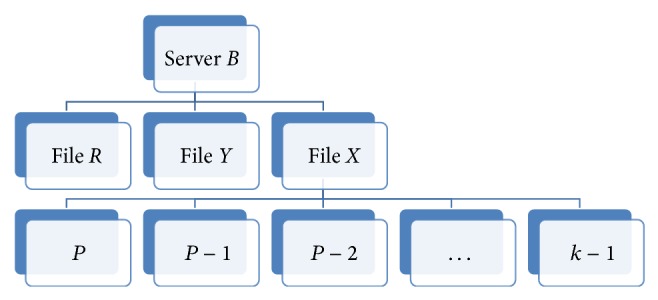
Cloud Node* B* file structure.

As more requests are initiated for downloading a specific file, the controller will be able to remove some blocks from each partition on the servers. At the same time the download process will continue normally for future requests without noticing the partial replication. This will allow us to reduce the storage needed on the cloud servers, while achieving better levels of performance for the client. The Partial Replication Load Balancing Algorithm performs better as the number of downloads increases. This is because more information about the servers becomes available for the evaluation of their ability to get the different parts of the file. Figures 7 and 8 show how the file blocks are stored as file structures on the cloud servers to simplify the search process of the partitions' blocks for the client and, moreover, to secure the other files hosted by the server from being accessed by the wrong clients.

Pseudocodes 2 and 3 show the pseudocode for partition removal at the server level on the cloud. The main idea involves copying the file blocks into other smaller files based on BlockIDs in each server node. After that the original file is removed. To save partitions of the file on the servers, we first check the existing experience saved for that file. This experience is saved in a database that is available with the controller. All the rows saved for that specific file will be retrieved. Then for each server that provided a partition of the file, a directory will be created in that specific server containing the file ID. This is so that it becomes easier for the server to find the data of that file. When the directory is created, the method will check the database for which blocks were downloaded from that server. As long as there are blocks downloaded from the server, a small file containing the block IDs will be created in the directory and the binary will be written to the file starting from the first position of the block until the last position. The new file size will be matching the BlockSize. Therefore, we made sure that there is no additional storage needed when writing the partition of the original to the new small files.

**Figure 9:**
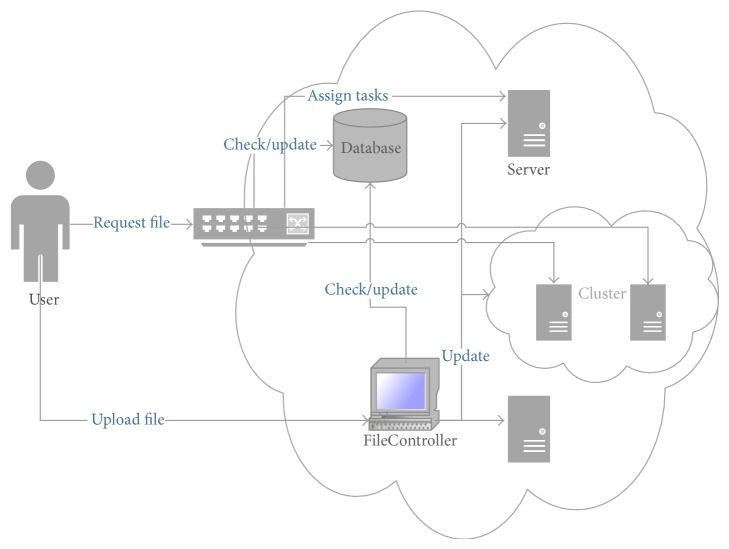
Solution components.

The components of the solution are shown in Figure 9. The main components are the client who initiates the request and sends it to the cloud, the load balancer which checks the file download experiences from the database and assigns tasks to the Cloud servers, the servers which process the requests, and the file controller which partitions the files at the storage level after checking the experiences of the file downloads. Our technique worked well when all servers are running well and processing their assigned tasks. However, we are in the process of analyzing the situation when one or multiple servers fail. In this case, we need to have more replicated file partitions as backup. This will be implemented in our future work.

## 5. Simulation

In this section we discuss the evaluation of our algorithm in terms of storage optimization and performance enhancement compared to other relevant algorithms.

### 5.1. Storage Simulation

First, we tested our storage optimization against other techniques, and we selected two load balancing algorithms. We then evaluated the storage optimization of our partial replication technique against the others in terms of saved storage space. The algorithms we selected are MapReduce and the ant colony algorithm. We also used different file sizes 100 MB, 500 MB, 1 GB, 2 GB, and 3 GB. We wanted large file sizes so that the storage difference can be demonstrated more clearly.

**Figure 10:**
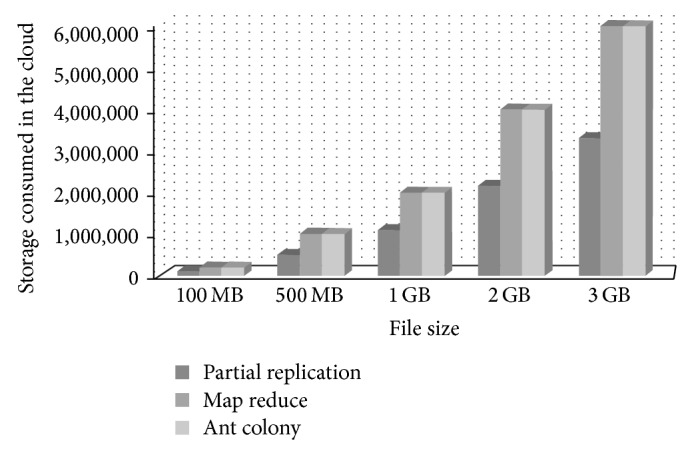
Storage consumption comparison using 2 cloud servers.

Our algorithm proved to reduce the need of storage needed on the cloud servers by at least 50% of the total storage. The difference is because we partition the file and save only the blocks that are usually provided by that server and we remove the other partitions. The other partitions are available on other servers. Moreover, saving about 3 GB of storage when the file size is very large is a very promising result, especially if many of the files on the cloud are large files. The test case we used is when having only two servers processing the file on the cloud. However, if there are more servers, the replicated blocks will increase based on the number of servers as described earlier. The differences in storage use among the three techniques are shown in Figure 10.

When testing the same algorithms using 4 cloud servers. The difference increased further, even when the number of replicated blocks in the partial replication algorithm increased. However, the difference between our algorithm and the others is much higher as they all use full replication of data.

**Figure 11:**
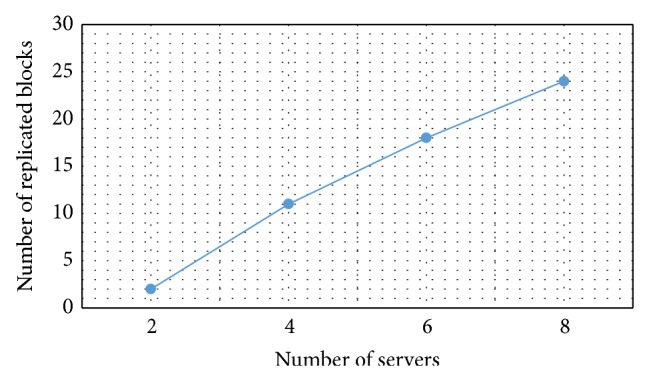
Effect of number of servers on blocks replication.

In addition to testing the performance of the algorithm while increasing the number of servers, we also simulated the storage consumed when the number of servers increases. To do that, we simulated a download for a 100 MB file using 2, 4, 6, and 8 servers. Then we ran our algorithm for optimizing the storage on the servers.  Figure 11 shows the amount of storage consumed for each group of servers. We noticed from the results that the storage consumed will increase when the number of servers increases. This is because for each dual servers working on a partition, there are blocks replicated and those are the blocks where the two servers meet in the download process. The percentage of the replicated blocks is very low compared to other full replication techniques. The other full replication techniques will double the storage consumed as the number of servers increases.

**Figure 12:**
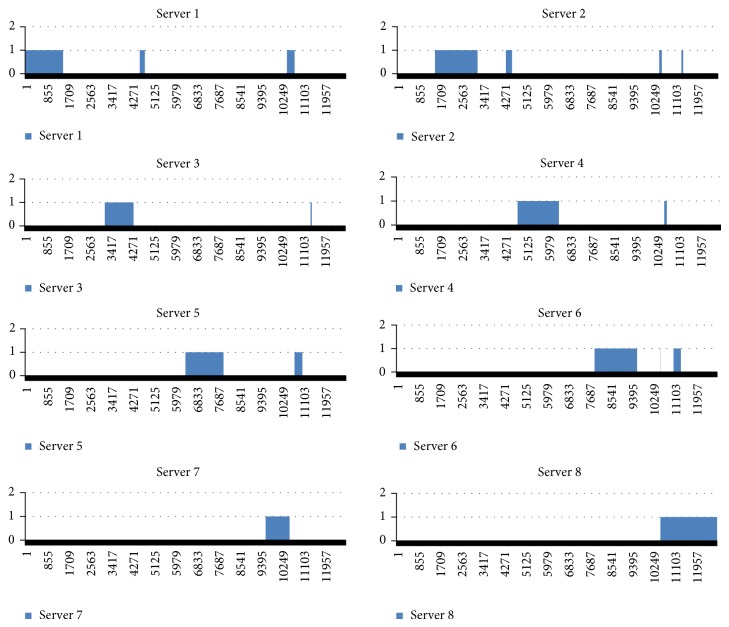
104 MB file partitions stored in 8 servers based on experience.

When downloading a file of size 100 MB by eight cooperative servers, we noticed that the number of replicated blocks was 25. First, the block size was determined using (2). Since the original file size is 104,857,600 bytes. The block size is 8192 bytes and therefore the total number of blocks is 12800.  Figure 12 shows the blocks saved in each of the eight servers based on the download experience.

### 5.2. Performance Simulation

**Figure 13:**
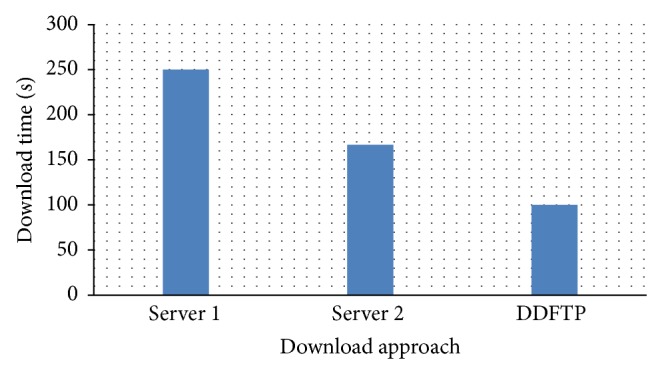
Effect of number of servers on blocks replication.

To evaluate the proposed algorithm, let us consider a data set initially replicated on two cloud servers at different locations. The size of the data set is 50 MB. As this data is replicated on both servers, a total of 100 MB is used. The data set is divided into 5000 blocks of 10,000 bytes each. Let us assume that the average download speed from the first server to different clients over the Internet is 20 blocks/second with a minimum of 15 blocks/second and a maximum of 20 blocks/second. The average download speed from the second server to different clients is 30 blocks/second with a minimum of 25 blocks/second and a maximum of 30 blocks/second. The average download times using only the first server, only the second server, and both servers using DDFTP are shown in Figure 13.

**Figure 14:**
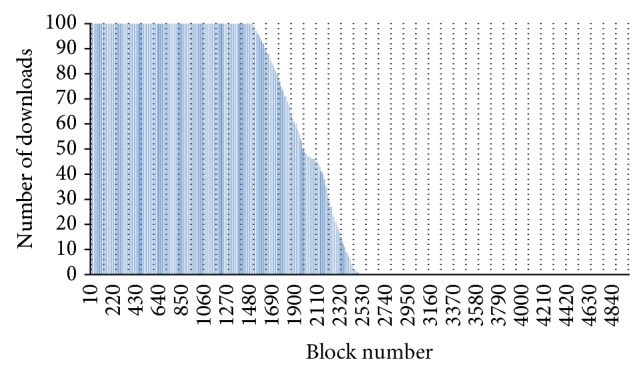
Blocks download from Server 1.

**Figure 15:**
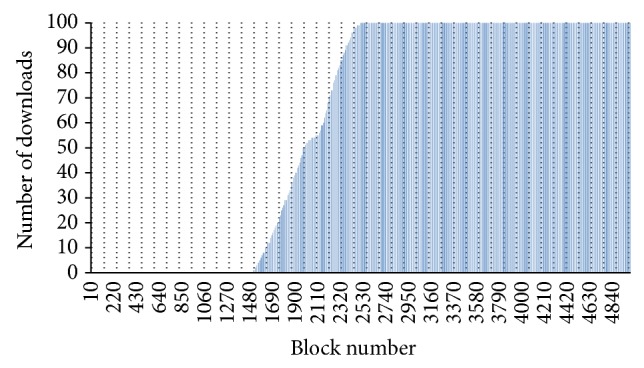
Blocks download from Server 2.

After 100 downloads of the data set from different clients, the number of downloads for each block is shown in Figure 14 for the first server and in Figure 15 for the second server. The first server never downloaded blocks higher than block number 2500 while the second server never downloaded any blocks lower than block number 1500. The main reason to getting these numbers is based on two cases. The first case is when the first server was downloading with its maximum speed while the second server was downloading with its minimum speed. Thus, the maximum block number that the first server downloaded is block number 2500 as both servers will be downloading an average of 25 blocks/second. The second case is when the first server was downloading with its minimum speed while the second server was downloading with its maximum speed. Thus, the minimum block number that the second server will download is block number 1500 as the speed of the first server is 15 blocks/second while the speed of the second server is 35 blocks/second. Using the approach developed in this paper, it is possible to remove the last 2500 blocks from the first server and the first 1500 blocks from the second server without affecting the parallel download operations and without increasing the download time.

**Figure 16:**
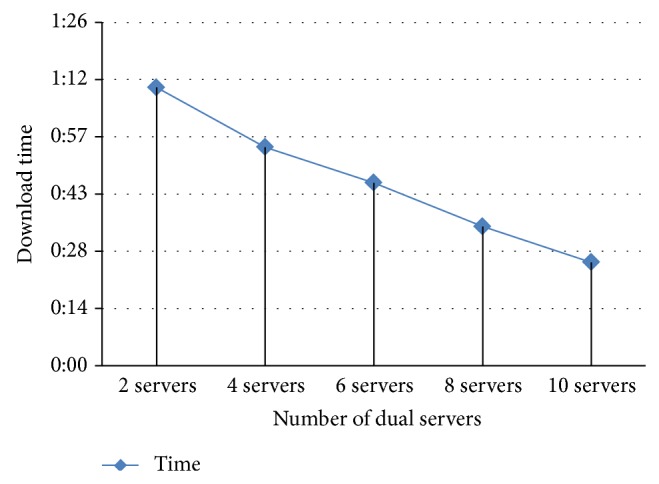
Effect of increasing the number of servers on download time.

For more performance evaluation, we simulated the file download speed using different numbers of dual servers. We first conducted an experiment using only two servers. Then, we conducted more experiments by increasing the number of servers to 4, 6, 8, and 10 servers. We noticed that the time needed to process the request reduces every time we increase the number of servers.  Figure 16 shows the finishing time of each request done by the number of servers specified. However, as discussed earlier, in a real cloud environment the speed and load on the servers change every second. Therefore, we are trying to come up with a prediction algorithm of the servers' behavior to calculate the optimal number of servers needed for each file and automate the download process and storage usage.

**Figure 17:**
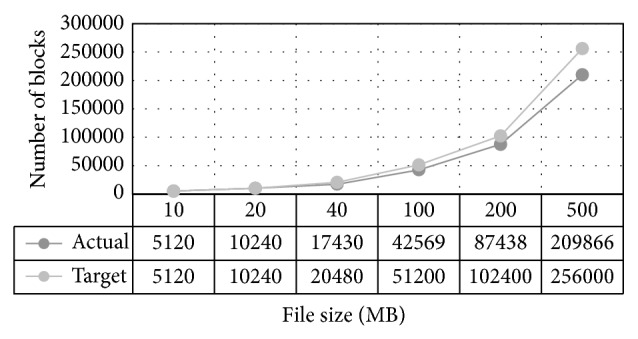
Error rate caused by the database server in case of larger number of connections.

The issue of the number of connections will mostly appear at the database server side. This is because a large number of connections could affect any database server and result in an inefficient performance. This is why we make sure that the blocks are of a size big enough to reduce the number of communication with the database server whenever a block is added to the file. The approach is to split the file into multiple blocks and then save them as separate files in the target folder on the hosting server. It will also save each block record in the database so that we can target any action taken to the block such as download or delete.  Figure 17 shows the error rate in case the number of connections of the database server were not considered. In case the number of connections were not considered, the database server will crash at some point. It usually recovers and saves the rest of the blocks but we noticed it never saved all the correct rows. We also noticed that as the file size increases, the error rate between the actual rows saved and the real value that should be saved increases. To solve this issue, we consider the number of hosting servers (NOS) and the number of available database server connections (NOC) when calculating the block size of the target file.

## 6. Conclusion

Storage optimization is an important issue as the demand for storage space especially for big data is rapidly increasing. However, data replication is also important for various reasons such as increasing reliability, fault tolerance, and enhanced performance and also for backup purposes. Yet replication increases the demand on storage significantly, which increases the cost of data storage and related services. In this paper, we presented an enhancement to the idea of partial replication load balancing algorithm. We used this algorithm based on a previous analysis of several well-known algorithms for load balancing on the cloud. We improved the dual direction algorithm (DDFTP) by allowing the storing of only parts of the files on each cloud server based on download history of these parts. Generally, we were able to reduce the number of partitions stored on each server and, as a result, we reduced the storage requirements for the replicas. The experimental results show the overall enhancements in storage use compared to the other load balancing algorithms. In addition, we were able to show that strategically removing some partitions from each replica based on past download experiences does not affect the performance or reliability of the system. Unfortunately this algorithm does not take into account the possibility of server failures. In the future, we are planning to improve this algorithm by adding efficient mechanisms to overcome the failure of any of the cloud servers involved. Furthermore, we plan to more thoroughly investigate and try to resolve the fault tolerance issues involved effectively and efficiently.

## Figures and Tables

**Pseudocode 1:**
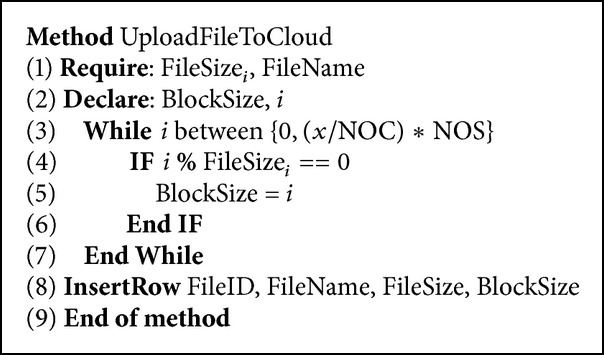
Pseudocode to find block size.

**Pseudocode 2:**
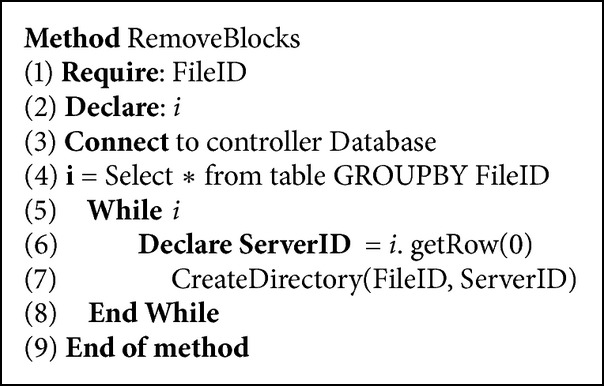
Remove block method pseudocode.

**Pseudocode 3:**
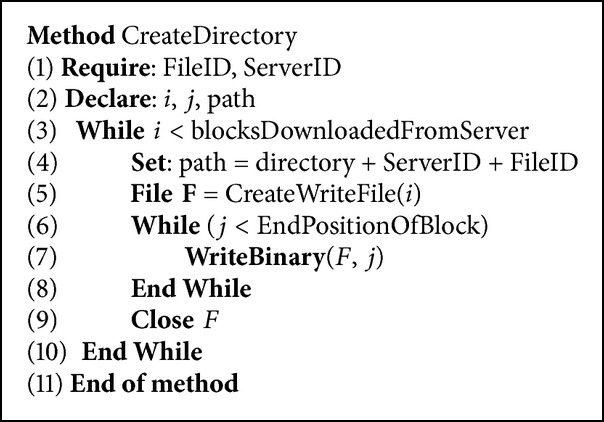
Create directory pseudocode.

**Table 1:** Current industry applications of DaaS.

Application	Free storage	Premium storage	Market share
Dropbox	2 GB	200 GB	47.9%
Google Drive	7 GB	200 GB	16.5%
iCloud	15 GB	50 GB	10.5%
OneDrive	5 GB	200 GB	9.3%

**Table 2:** Comparison of load balancing algorithms.

Algorithm	Replication	SOF	Network overhead	Spatially distributed	Fault tolerance
INS	Partial	Yes	Yes	Yes	No
ESWLC	Full	No	Yes	Yes	Yes
CLBDM	Full	Yes	Yes	Yes	No
Ant colony	Full	No	Yes	No	Yes
Map reduce	Full	No	Yes	Yes	Yes
VM	Full	Yes	Yes	No	Yes
DDFTP	Full	No	No	Yes	Yes
LBMM	Full	No	No	Yes	Yes
